# Outcome Measures to Assess the Effectiveness of Exercise Interventions on Chemotherapy-Induced Peripheral Neuropathy (CIPN): A Scoping Review

**DOI:** 10.3390/curroncol33040231

**Published:** 2026-04-20

**Authors:** Trei R. Lindstrom, Joanna F. Parkinson, Kerry S. Courneya, Margaret L. McNeely

**Affiliations:** 1 Faculty of Rehabilitation Medicine, University of Alberta, 2-50 Corbett Hall, Edmonton, AB T6G 2G4, Canada; trei@ualberta.ca (T.R.L.); jfparkin@ualberta.ca (J.F.P.); 2 Faculty of Kinesiology, Sport, and Recreation, University of Alberta, 1-113 University Hall, Edmonton, AB T6G 2H9, Canada; kerry.courneya@ualberta.ca; 3 Department of Oncology, Cancer Care Alberta, Edmonton, AB T6G 1Z2, Canada

**Keywords:** chemotherapy-induced peripheral neuropathy, exercise interventions, outcome measures, scoping review

## Abstract

Chemotherapy-induced peripheral neuropathy (CIPN) is a common side effect of neurotoxic chemotherapy that can affect functioning and quality of life. Currently, duloxetine is the only recommended agent to treat painful CIPN; however, no effective pharmacological treatments have been approved for the prevention or cure of CIPN, highlighting the need to understand non-pharmacological strategies such as exercise. Given significant heterogeneity in the CIPN outcome measures chosen across studies, this scoping review aimed to identify the outcome measures used to evaluate the effectiveness of exercise interventions as a potential countermeasure for CIPN. Following the Arksey and O’Malley framework refined by Levac and colleagues, and the PRISMA-ScR guidelines, four databases were searched, and 20 studies were included in the review. Data were abstracted on study characteristics, cancer and chemotherapy factors, exercise prescription, outcome measures, and CIPN-related findings. Outcome measures varied widely across studies, encompassing various patient-reported, clinical, and functional measures. The most common patient-reported, clinical, and functional measures were the EORTC QLQ-CIPN20, vibration sensation, and maximal isometric strength, respectively. No study satisfied the components of the core outcome measure set proposed by Park and colleagues, limiting cross-study comparisons. These findings underscore the need for standardized CIPN outcome measures in future exercise studies to strengthen evidence synthesis and inform clinical practice.

## Simple Summary

Chemotherapy can cause nerve damage that leads to pain, tingling, numbness, and problems with balance and daily activities. There are no known drugs to treat these symptoms, yet exercise is becoming a promising strategy for managing them. Studies use many different measures to assess the effectiveness of exercise, making it difficult to compare studies and determine which measures work best. In this review, we explored the various measures studies have used to evaluate the effectiveness of exercise in reducing nerve damage caused by chemotherapy. We found substantial variability in how symptoms, functioning, and quality of life were measured across studies, and no study used the full set of measures recommended by experts. While some studies found exercise is effective in reducing symptoms, greater consistency in the measurement approach is needed. This review may inform future studies in selecting standardized outcome measures.

## 1. Introduction

Chemotherapy-induced peripheral neuropathy (CIPN) is a common side effect of neurotoxic chemotherapeutic agents used in cancer treatments [1]. Standard chemotherapy drug classes known to cause CIPN include taxanes (e.g., paclitaxel and docetaxel), platinum-based (e.g., oxaliplatin and cisplatin), and vinca alkaloids (e.g., vincristine and vinblastine), resulting in CIPN incidences of 11% to 87%, 49% to 100%, and 60%, respectively [1]. The pooled prevalence of moderate-to-severe or painful CIPN is currently estimated at 47.76%, with higher rates in individuals receiving taxanes and platinum-based agents [2]. Additionally, the greatest prevalence of moderate-to-severe CIPN or painful CIPN based on primary cancers is in individuals with breast cancer, multiple myeloma, and lung cancer [2]. CIPN symptoms encompass both sensory and motor symptoms, which can be classified as either positive or negative symptoms [3]. Positive CIPN symptoms exacerbate sensory disturbances, including tingling, pain, and altered sensitivity to hot and cold temperatures [1,3]. Negative CIPN symptoms represent loss of sensation and function, including numbness and difficulty with fine motor movements [1,3]. Thus, CIPN has the potential to negatively impact an individual’s health-related quality of life (HRQL), interfere with physical functioning, and impact chemotherapy adherence [1,4]. Despite the common occurrence of CIPN, there are no established, effective pharmacological agents approved for its prevention or cure [4]. Duloxetine is currently the only recommended agent for treating painful CIPN, supported by moderate-strength recommendations based on intermediate-quality evidence [4]. This underscores the need for further exploration into non-pharmacological and lifestyle interventions aimed at mitigating CIPN symptoms and improving function for individuals with cancer [5].

Exercise has emerged as a potential lifestyle intervention for preventing and treating CIPN symptoms [1]. Most exercise interventions for CIPN symptoms employ a combination of exercise modalities—namely aerobic, resistance, and balance training [6]. CIPN and exercise studies have demonstrated that exercise is feasible and can provide significant benefits in managing symptoms, including reductions in pain and numbness, as well as improvements in balance and QOL [6,7]. While the underlying mechanisms are unclear, exercise interventions may alleviate CIPN symptoms by positively modulating neurotrophins and reducing inflammation [7].

Studies investigating exercise for CIPN have used a range of outcome measures to evaluate symptoms and physical functioning, including patient-reported and objective outcome measures. A systematic review and consensus expert opinion [8] proposed a set of core outcome measures for CIPN and exercise-related rehabilitation studies. This core set includes measures of CIPN symptoms, CIPN impact, and balance and gait [8]. Additional important domains include measures of strength and physical fitness, neurological examination, and quality of life, along with outcomes such as function, ataxia, falls, and pain [8]. However, heterogeneity in outcome selection across studies limits cross-study comparison and consensus [8]. In addition, although evidence supports combining patient-reported and objective outcomes [8], many studies rely solely on patient-reported measures, which increases the potential for self-report bias and reduces comparability [9].

The purpose of this scoping review is to identify and synthesize existing literature on outcome measures used in studies examining the effectiveness of exercise as a potential countermeasure for CIPN. Specific objectives are (1) to explore the type, frequency of administration, and findings of outcome measures; and (2) to describe chemotherapy factors, exercise prescription characteristics, and the included cancer populations. The overall aim is to highlight potential gaps in current research and make recommendations concerning the types, frequency, and timing of CIPN measures for specific exercise interventions.

## 2. Materials and Methods

This scoping review follows a framework proposed by Arksey and O’Malley [10] and refined by Levac and colleagues [11]. Reporting in this review follows the PRISMA Extension for Scoping Reviews (PRISMA-ScR) [12]. The protocol for this scoping review was registered with Figshare and is accessible at https://doi.org/10.6084/m9.figshare.31982271. A scoping review was chosen to explore the current state of chosen outcome measures for examining the effects of exercise on CIPN, and to address potential literature gaps.

### 2.1. Stage One: Identifying the Research Question

Our chosen research question was: What outcome measures have been used to evaluate the effectiveness of exercise interventions as a potential countermeasure for CIPN in patients who are undergoing/have undergone neurotoxic chemotherapy? Specifically, we sought to identify the type, frequency, and findings of the outcome measures. Furthermore, we examined participants’ chemotherapy status and cancer type, as well as the exercise prescription parameters applied in the studies. For this review, exercise was defined as “planned, structured, and repetitive bodily movement performed to improve or maintain one or more components of physical fitness” [13]. For the purpose of this review, studies involving alternative forms of exercise (e.g., yoga, dance) were excluded as these interventions do not typically specify quantifiable exercise prescription parameters, such as intensity.

### 2.2. Stage Two: Identifying Relevant Studies

The search strategy was developed with a health sciences librarian and conducted in four electronic databases (CINAHL, Embase, Medline, and Scopus). Each search encompassed all potential date ranges, and no language limits were applied. The final searches for each database were made on 19 January 2026. Details of each search are provided in Supplementary Materials.

Published papers were deemed eligible if they employed a randomized controlled trial (RCT) design including protocols and secondary analyses. Additional inclusion criteria required that studies: (1) involve human adults (aged 18+) with or at risk of CIPN that are set to undergo, are currently undergoing, or have completed chemotherapy, (2) list CIPN as a primary or secondary outcome, (3) employ only exercise in the intervention, (4) and implement one or a combination of patient-reported or objective outcome measures of CIPN.

Studies were excluded if they implemented (1) combined interventions (e.g., nutrition/pharmacological agents + exercise) unless the effect of exercise could be isolated, (2) recreational activities such as yoga, tai chi, stretching, qigong, Pilates, dance, and sport-based interventions, (3) non-exercise therapeutic modalities (e.g., vibration, sensorimotor, ultrasound) unless the effect of exercise could be isolated, (4) children, adolescents, or adult survivors of childhood cancer, and (5) animal studies.

### 2.3. Stage Three: Study Selection

The citations from each search were uploaded to Covidence systematic review software (Veritas Health Innovation, Melbourne, Australia) to complete the title/abstract screening, full-text review, and data extraction processes. All duplicates were removed. Two reviewers (TRL and JFP) independently screened each title/abstract. The full-text review also included an independent review process conducted by the two reviewers (TRL and JFP) of all remaining citations. Disagreements were resolved through consensus discussions between the two independent reviewers after both title/abstract screening and full-text review.

### 2.4. Stage Four: Charting the Data

A data extraction template was consistently used to identify all relevant information from each study. The extraction procedure involved identifying the following: study characteristics (title, author, publication year, study country, study design, and study purpose); study population characteristics (participant demographics and medical characteristics); exercise prescription parameters; outcome measures (name, type, and timing); and primary results. Data extraction was performed independently by two reviewers (TRL and JFP), and any discrepancies were resolved through consensus discussion.

### 2.5. Stage Five: Collating, Summarizing, and Reporting the Results

To provide a comprehensive overview of the included studies, we collated, summarized, and reported study characteristics (i.e., study country, study design), participant characteristics (i.e., age, sex, cancer type, chemotherapy type and status), exercise intervention details (i.e., exercise class, frequency, duration, setting), outcome measures used, and findings related to use of these outcome measures.

## 3. Results

### 3.1. Study Selection

**Figure 1:**
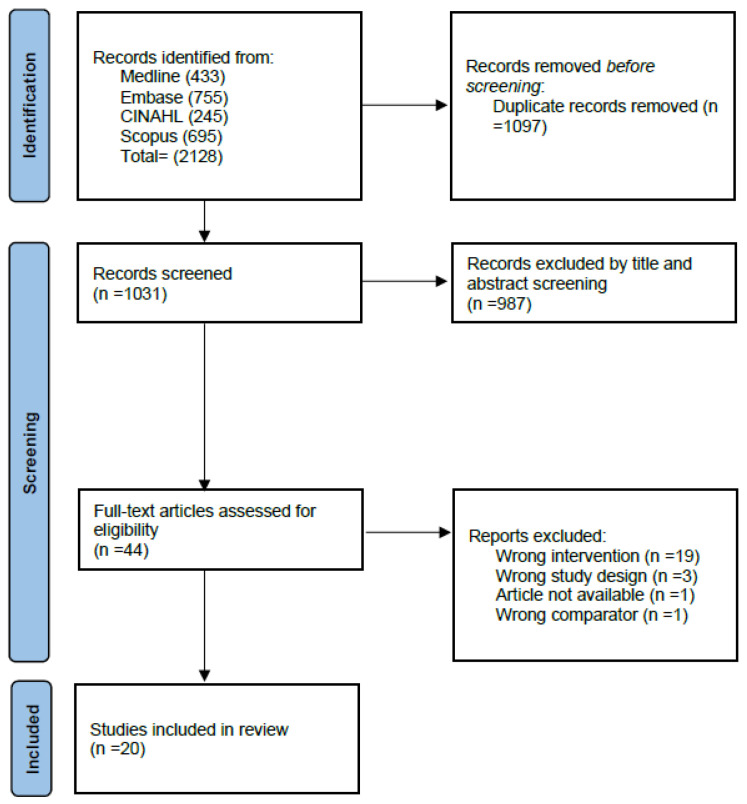
PRISMA flow diagram.

A total of 2128 studies were retrieved, and 1097 duplicates were removed. The remaining 1031 (48.4%) studies were screened for eligibility through title/abstract screening. Inter-rater reliability was deemed “moderate” [14] with a Cohen’s Kappa of 0.72 for title/abstract screening. Following title/abstract screening, 44 studies were included in the full-text review. Inter-rater reliability was deemed “weak” [14] with a Cohen’s Kappa of 0.55 for full-text review. In total, 24 studies were excluded in the full-text review. The most common exclusion reason was an incorrect intervention (i.e., the intervention was not limited to exercise alone) (Figure 1).

### 3.2. Study Characteristics

A total of 20 studies published between 2014 and 2025 were included in this review (Table 1). Seven studies were from Germany [15,16,17,18,19,20,21], six were from the United States [22,23,24,25,26,27], two were from Turkey [28,29], with one study each from Denmark [30], Canada [31], India [32], Spain [33], and the Republic of Korea [34]. Thirteen studies were RCTs [15,16,17,18,19,21,23,26,28,29,31,32,34], four were secondary analyses of RCTs [20,22,25,30], and three were RCT study protocols [24,27,33].

**Table 1:** Study characteristics and findings.

Study/Country	Study Design	Cancer Type(s)	Chemotherapy Agent(s)	Chemotherapy Status	Exercise Type(s)	Assessment Timepoints	Main CIPN-Related Findings
Henke et al., 2014 [15], Germany	RCT	Lung (n = 29, 100%)	Platinum-based	During	Resistance, aerobic	Baseline, post-intervention	Exercise group showed significant improvement in EORTC QLQ-C30/LC13 scores
Zimmer et al., 2018 [16], Germany	RCT	Colorectal (n = 30, 100%)	Platinum-based	During & following	Balance, aerobic, resistance	Baseline, post-intervention, 4-week follow-up	CIPN worsened in control group but remained stable in exercise group
Kleckner et al., 2018 [22], USA	Secondary analysis of RCT	Breast (n = 281), Lymphoma (n = 18), Colon (n = 18), Lung (n = 12), Other (n = 26)	Multiple: Taxane, platinum-based, vinca alkaloid	During	Aerobic, resistance	Baseline, post-intervention	Exercise reduced hot/cold sensitivity in hands/feet and numbness and tingling; effects were stronger in males, older adults, and individuals with breast cancer
Bland et al., 2019 [31], Canada	RCT	Breast (n = 27, 100%)	Taxane	During (IE) and following (DE)	Aerobic, resistance, balance	Prior to chemotherapy, after 3 taxane cycles and 0–3 days before the fourth and final taxane cycle, at the end of chemotherapy, 10–15 weeks after chemotherapy	No group differences in EORTC QLQ-CIPN20 scores; exercise group had reduced numbness, better vibration sense, and higher QOL mid-treatment; no group differences post-chemotherapy
Stuecher et al., 2019 [17], Germany	RCT	Pancreatic (n = 9), Gastric (n = 6), Colon (n = 23), Esophageal (n = 4)	Not reported	During	Aerobic	Baseline, 1 day before first chemotherapy (T0), after 4–6 weeks of chemotherapy (before third cycle) (T1), after 12 weeks of chemotherapy (T2)	Exercise improved postural sway and body composition; no group differences in neuropathy, strength, or gait
Kneis et al., 2019 [18], Germany	Results of RCT	Breast (n = 26), Colorectal (n = 27), Gynecological (n = 7), Upper GI (n = 4), Non-small cell lung (n = 2), Non-Hodgkin lymphoma (n = 10), Multiple myeloma (n = 2)	Not reported	Following	Aerobic, balance	Baseline, post-intervention	No group differences in postural sway (primary outcome); per-protocol analysis showed improved balance and reduced motor symptoms in adherent exercisers
Dhawan et al., 2020 [32], India	RCT	Ovarian (n = 28), Cervical (n = 8), Lung (n = 5), Retromolar trigone (n = 1), Parotid ductal carcinoma (n = 1), Base of tongue (n = 1)	Multiple: Taxane, platinum-based	During	Resistance, balance	Baseline, post-intervention	Exercise significantly reduced neuropathic pain and improved QOL
Müller et al., 2021 [19], Germany	RCT	Breast (n = 121), Pancreatic (n = 9), Prostate (n = 5), Stomach (n = 5), Esophageal (n = 4), Colon (n = 4), Brain (n = 3), Ovarian (n = 3), Tongue (n = 2), Rectal (n = 2), Bladder (n = 1), Anal (n = 1), Lung (n = 1), Cervix uteri (n = 1), Malignant neoplasm without specification of site (n = 1)	Multiple: Taxane, platinum-based, vinca alkaloid	Before and during	Resistance	Baseline, 3 weeks after chemotherapy completion (post0), 3 months after post0, 6 months after post0	No CIPN differences between groups in ITT analysis; adherent exercisers had less subjective lower-body sensory symptom progression during chemotherapy
Waibel et al., 2021 [20], Germany	Secondary analysis of RCT	Colorectal (n = 12), Breast (n = 11), Gynecological (n = 2), Upper gastrointestinal (n = 2), Non-Hodgkin lymphoma (n = 4)	Not reported	Following	Aerobic, balance	Baseline, post-intervention	Exercise reduced spontaneous sway and improved sensory-motor control; group differences in postural reaction timing
Kanzawa-Lee et al., 2022 [23], USA	RCT	Colon (n = 22), Rectal/colorectal (n = 16), Pancreatic (n = 18), Esophageal (n = 1)	Platinum-based	During	Aerobic	Baseline, week 8 (6th chemotherapy infusion)	No exercise effects on sensory or motor CIPN symptoms; symptom severity increased over time in both groups
Teran-Wodzinski et al., 2022 [24], USA	RCT	Breast (n = NR, 100%)	Taxane	Following	Gait/balance, resistance	Baseline, post-intervention	Not applicable (protocol only)
Şimşek & Demir, 2021 [28], Turkey	RCT	Breast (n = 90, 100%)	Taxane	During	Resistance, balance	Baseline, post-intervention	Exercise reduced hand and foot numbness compared to cold application and control groups
Martin-Sanchez et al., 2024 [33], Spain	RCT	Colorectal (n = NR, 100%)	Not reported	During	Resistance, aerobic	Baseline, post-intervention	Not applicable (protocol only)
Cao et al., 2023 [25], USA	Secondary analysis of RCT	Ovarian (n = 134, 100%)	Multiple: Taxane, platinum-based	During	Aerobic	Baseline, 6 months	Exercise significantly reduced FACT/GOG-Ntx scores compared to control
Kleckner et al., 2024 [26], USA	RCT	Breast (n = 8), Colon (n = 3), Myeloma (n = 3), Pancreatic (n = 2), Bladder (n = 1), Esophageal (n = 1), Prostate (n = 1)	Multiple: Taxane, platinum-based	During	Aerobic, resistance	Baseline, Mid-intervention, post-intervention	Exercise demonstrated moderate to large effect sizes on CIPN symptoms/signs; no significant group differences; both groups worsened in EORTC QLQ-CIPN20 scores
Hwang et al., 2025 [34], Republic of Korea	RCT	Colorectal (n = 34, 100%)	Platinum-based	During	Aerobic, balance, resistance	Baseline, 4 weeks post-intervention, 6 weeks post-intervention	Exercise app group significantly reduced neuropathy symptoms and interference with activities, and improved QOL compared to control
Manuweera et al., 2025 [27], USA	RCT	Breast (n = NR), Gastrointestinal (n = NR)	Multiple: Taxane, platinum-based	During	Aerobic, resistance	Baseline, mid-intervention, post-intervention, 12-week follow-up	Not applicable (protocol only)
Ozdemir & Arslan, 2025 [29], Turkey	RCT	Breast (n = 82, 100%)	Taxane	During	Aerobic	Baseline, week 4, week 8, post-intervention	Exercise significantly reduced CIPN symptoms compared to control group; CIPNAT & SF-MPQ scores significantly worsened in control group compared to intervention group
Dalferth et al., 2025 [21], Germany	RCT	Breast (n = 66, 100%)	Taxane	During	Resistance	Baseline, week 6, post-intervention	No significant differences between groups; vibration sensation decreased less in intervention group, and motor symptoms worsened in control group
Bloch et al., 2025 [30], Denmark	Secondary analysis of RCT	Lung (n = 218, 100%)	Multiple: Taxane, platinum-based, vinca alkaloid	During	Aerobic, resistance	Baseline, post-intervention	CIPN symptoms remained unchanged in intervention group, yet significantly increased in control group

### 3.3. Sampe Sizes

Sample sizes of the included studies ranged from 19 [26] to 355 [22], with a median sample size of 45. Age statistics were inconsistently reported; however, the most common median ages reported were between 50 and 60 years. Across the 15 studies that included participant sex statistics [16,17,18,19,20,21,22,23,25,26,29,30,31,32,34], the majority of participants were female (77%). The three most common cancer types in the included studies were breast, lung, and colorectal cancers. Four studies included only individuals with breast cancer [21,28,29,31]. Two studies exclusively included participants with colorectal cancer [16,34]. As well, two studies included only individuals with lung cancer [15,30]. The remaining single-cancer-type studies included one for ovarian [25]. The most reported chemotherapy agents were taxane-based, with five studies [21,24,28,29,31] including participants who had undergone taxane-based chemotherapy. Four studies [15,16,23,34] included participants who had undergone only platinum-based chemotherapy. Seven studies [19,22,25,26,27,30,32] reported combinations of taxanes, platinum-based, and vinca alkaloid chemotherapy agents. The remaining four studies did not report the use of chemotherapy agents [17,18,20,33]. The majority (14/20) of studies included participants who were undergoing chemotherapy [15,17,21,22,23,25,26,27,28,29,30,32,33,34]. Three studies [18,20,24] included participants who had completed chemotherapy. Two studies [16,31] included participants who completed chemotherapy during the intervention period. One study [19] included participants at risk of CIPN before undergoing chemotherapy.

### 3.4. Exercise Interventions

The most common exercise modality employed by six studies [15,22,26,27,30,33] was combined aerobic + resistance training. Four studies [17,23,25,29] focused solely on aerobic exercise. Three studies employed combinations of aerobic + resistance + balance [16,31,34], and three involved resistance + balance [24,28,32]. Two studies employed a combination of aerobic + balance exercise [18,20], and two involved resistance training alone [19,21]. While inconsistently reported, individual exercise session durations ranged from 10 min [23,29] to 1.5 h [30]. The duration of exercise programming ranged from six weeks [22,34] to six months [25]. Eleven interventions were unsupervised, home-based exercise programs [17,22,23,24,25,26,27,28,29,32,34], with one delivering exercise through an app-based program [34]. Six interventions [15,16,18,20,21,30] were supervised programs, while three interventions [19,31,33] were a combination of supervised and home-based programs. Adherence rates were inconsistently reported across studies, with reported rates ranging from 33% [19] to 90% [18].

### 3.5. Outcome Measures Results (Table 2)

Of the patient-reported CIPN symptom-related outcome measures, the most commonly implemented measure (8/20) was the European Organization for Research and Treatment of Cancer Quality of Life Questionnaire CIPN 20 subscale (EORTC QLQ-CIPN20) [18,20,21,23,26,27,31,33]. Four studies implemented the Chemotherapy-Induced Peripheral Neuropathy Assessment Tool (CIPNAT) [28,29,32,34]. Three studies implemented the Functional Assessment of Cancer Therapy/Gynecologic Oncology Group—Neurotoxicity (FACT/GOG-Ntx) [16,18,25]. Single studies used the following: 0–10 scale to measure numbness and tingling and hot/cold sensitivity in the hands/feet [22], a structured pro forma (Performa as stated by the authors; interpreted as pro forma) for primary assessment of CIPN [32], the Leeds Assessment of Neuropathic Symptoms and Signs (LANSS) Pain scale [32], the European Organization for Research and Treatment of Cancer Quality of Life Questionnaire CIPN 15 subscale (EORTC QLQ-CIPN15) [32], the Functional Assessment of Cancer Therapy—Taxane (FACT-Taxane) additional concerns subscale [24], a daily diary to track CIPN symptoms [27], a cold stimulation task [27], the Pain Catastrophizing Scale (PCS) [27], and the Short form McGill Pain Questionnaire (SF-MPQ) [29]. Three studies [15,17,30] did not implement a patient-reported CIPN symptom-related outcome measure.

Among the clinical CIPN outcome measures, the most commonly used measure (5/20) was vibration sensation [17,18,20,21,31]. Three studies implemented a tactile sensitivity test using monofilaments [21,26,27]. Two studies utilized nerve conduction velocity (NCV) testing [24,32]. Single studies implemented the following instrumental/clinical measures: Total Neuropathy Score (TNS) [28], reduced Total Neuropathy Score (TNSr) [19], pinprick [31], and magnetic resonance imaging (MRI) brain imaging [27]. Nine studies [15,16,22,23,25,29,30,33,34] did not include an instrumental/clinical CIPN outcome measure.

Of the functional assessments of CIPN impact, the most commonly used measure (5/20) was maximal isometric voluntary strength/force [17,19,24,26,27]. Four studies implemented the 6-Minute Walk Test (6MWT) [16,26,27,33]. Two studies each measured unipedal stance time and vertical jump [18,20], postural sway [17,18], direct measures of cardiorespiratory fitness (including combinations of peak oxygen uptake (VO2peak), maximal aerobic power (Pmax), individual anaerobic threshold (IAT)) [18,30], and the Fullerton Advanced Balance Scale (FABS) [21,27]. Single studies implemented the following functional assessments: Gleichgewichtstest Rehabilitation Compliant Floor Reaction Force Platform Balance Test (GGT-Reha) [16], hypothetic one-repetition maximum (h1RM) [16], Sport Physical Performance Battery (SPPB) [17], postural control [19], Fall Efficacy Scale-International (FES-I) [19], number of falls [19], spontaneous sway [20], perturbed stance [20], the Ambulatory Parkinson’s Disease Monitoring (APDM) Opal (Mobility lab v1, APDM, Inc., Portland, OR) inertial measurement units (IMU) gait assessment system [24], Sensory Organization Test (SOT) [24], isokinetic strength [26], activity tracking (Fitbit) [27], pedometry [29], a gait tracking chart [29], the Berg Balance Scale (BBS) [21], and the Romberg Test [21]. Eight studies [15,22,23,25,28,31,32,34] did not include a functional outcome measure of CIPN impact.

Nine studies [15,18,19,23,30,31,32,33,34] used the European Organization for Research and Treatment of Cancer Quality of Life Questionnaire Core 30 (EORTC QLQ-C30) to assess HRQOL. In addition to the EORTC QLQ-C30, one study [30] utilized the European Organization for Research and Treatment of Cancer Quality of Life Questionnaire—Lung Cancer 13 (EORTC QLQ-LC13) to measure disease- and treatment-related symptoms in individuals with lung cancer. Of the studies that did not implement the EORTC QLQ-C30, two utilized the FACT/GOG-Ntx [16,25] to measure HRQOL. The remaining nine studies [17,20,21,22,24,26,27,28,29] did not measure HRQOL. Several additional outcome measures were included, in addition to those previously mentioned. Two studies [27,33] utilized the Hospital Anxiety and Depression Scale (HADS). One study [17] included the Mini Nutritional Assessment (MNA) and Bioelectrical Impedance Analysis (BIA). One study [29] employed the Minimal Insomnia Symptom Scale (MISS) and the Functional Assessment of Cancer Therapy-Fatigue (FACT-F) scale. One study [32] utilized the Restless Leg Syndrome (RLS) pro forma (Performa as stated by the authors; interpreted as pro forma). One study [27] implemented resting physiological function (using an electrocardiogram), heartbeat detection tasks, blood draw, the Multidimensional Assessment of Interoceptive Awareness (MAIA) v2, symptom inventory, the Functional Assessment of Cancer Therapy—Cognitive Function (FACT-Cog), the Brief Fatigue Inventory (BFI), the Regulation of Emotion Systems Survey (RESS), the National Cancer Institute Fruit and Vegetable Screener (NCI FVS), and the Protein Screener 55+ (Pro55+). Lastly, a study [29] utilized the Eastern Cooperative Oncology Group (ECOG) Performance Scale.

All studies included multiple assessment timepoints. Most studies (11/20) performed outcome measure assessments at baseline (prior to intervention) and immediately following the intervention [15,18,20,22,23,24,25,28,30,32,33]. Three studies [17,21,26] performed outcome measure assessments at baseline, once during the intervention, and immediately following the intervention, while three studies [16,19,27] followed this same structure but performed (an) additional follow-up assessment(s) at four weeks [16], three weeks following chemotherapy completion (post0), three months following post0, six months following post0 [19], and at 12 weeks [27]. One study [29] assessed outcomes at baseline, twice during the intervention, and immediately after the intervention. Another study [34] assessed participants at baseline and conducted two follow-ups at four- and six-weeks post-intervention, with no assessment immediately following the intervention. Lastly, one study [31] assessed participants at baseline, after three taxane cycles and 0–3 days before the fourth and final taxane cycle, at the end of chemotherapy, and at a 10–15-week follow-up or following the intervention for the delayed exercise group.

Of the non-protocol studies, 8/17 reported a significant decrease in CIPN symptoms in the exercise group compared to the control [18,20,22,25,28,29,32,34]. Two of these studies [32,34] additionally reported improvements in HRQOL in favor of the exercise group, while another two of these studies [18,20] additionally reported improvements in functional outcomes in favor of the exercise group. Two studies [23,26] found no significant differences between the exercise and control groups, with CIPN severity increasing in both groups over time. Two studies [19,31] observed a significant reduction in CIPN symptoms during chemotherapy in the exercise group compared to the control; however, no differences in CIPN symptoms were observed following treatment. One study [16] reported stable CIPN symptoms and improved functional outcomes compared with the control group, which showed worsening in CIPN symptom severity. Similarly, another study [30] reported stable CIPN symptoms and decreased pain compared with the control group, which showed worsening in CIPN symptom severity. One study [17] found no differences in CIPN symptoms between the exercise and control groups, yet showed improvements in functional outcomes in the exercise group. Another study [15] reported improved HRQOL in the exercise group compared to the control group. The remaining study [21] reported stabilized vibration sense in the intervention group compared to the control group, yet no significant differences were found between groups. No adverse exercise effects were reported in any study.

**Table 2:** Outcome measure alignment with core set proposal by Park et al., 2022 [8].

		Core Set		Additional Important Domains	Further Outcomes/Domains
	CIPN Symptoms	Impact of CIPN	Balance and Gait		
Henke et al., 2014 [15]				EORTC QLQ-C30	
Zimmer et al., 2018 [16]	FACT/GOG-Ntx		GGT-Reha	h1RM; 6MWT	
Kleckner et al., 2018 [22]					Patient-reported 0–10 scale
Bland et al., 2019 [31]	EORTC-CIPN20		Vibration sensation; Pinprick	EORTC QLQ-C30	
Stuecher et al., 2019 [17]			Postural stability	SPPB; Maximal isometric force; Vibration sense	MNA; BIA
Kneis et al., 2019 [18]	EORTC-CIPN20; FACT/GOG-Ntx		Postural sway; Unipedal stance time	Vertical jump; Vibration sense; EORTC QLQ-C30; Cardiorespiratory fitness	
Dhawan et al., 2020 [32]		CIPNAT		NCV; EORTC QLQ-C30	Structured proforma for primary assessment of CIPN; RLS proforma; LANSS Pain Scale
Müller et al., 2021 [19]	EORTC-CIPN15		Postural control; FES-I; Number of falls	TNSr; Maximal isometric force; EORTC QLQ-C30	
Waibel et al., 2021 [20]	EORTC-CIPN20		Unipedal stance time; Spontaneous sway; Perturbed stance	Vibration sense; Vertical jump	
Kanzawa-Lee et al., 2022 [23]	EORTC-CIPN20			EORTC QLQ-C30	
Teran-Wodzinski et al., 2022 [24]	FACT-Taxane		ADPM Opal IMUs SOT	Isometric strength; NCV	
Şimşek & Demir, 2021 [28]		CIPNAT		TNS	
Martin-Sanchez et al., 2024 [33]	EORTC-CIPN20			EORTC QLQ-C30; 6MWT	HADS; FACT-F; MISS
Cao et al., 2023 [25]	FACT/GOG-Ntx				
Kleckner et al., 2024 [26]	EORTC-CIPN20			Tactile sensitivity test; 6MWT; Handgrip dynamometry; Isokinetic leg strength	
Hwang et al., 2025 [34]		CIPNAT		EORTC QLQ-C30	
Manuweera et al., 2025 [27]	EORTC-CIPN20; Daily diary; Cold stimulation; PCS		FABS	Activity tracker (fitbit); Tactile sensitivity; Handgrip dynamometry; 6MWT; MRI	ECG; Heartbeat detection; Blood draw; MAIA v2; HADS; Symptom Inventory; FACT-Cog; BFI; RESS; NCI FVS; Pro55+
Ozdemir & Arslan, 2025 [29]	SF-MPQ	CIPNAT		Pedometer; Gait tracking chart	ECOG Performance Scale
Dalferth et al., 2025 [21]	EORTC-CIPN20		FABS; BBS; Romberg Test	Vibration sense; Tactile sensitivity	
Bloch et al., 2025 [30]				EORTC QLQ-C30; EORTC QLQ-LC13; Cardiorespiratory fitness	

## 4. Discussion

Consistent with prior literature, our review revealed substantial heterogeneity in the outcome measures used across studies, with patient-reported, clinical, and functional assessments employed in different combinations [8,35,36]. Moreover, none of the studies satisfied the proposed core outcome set criteria (i.e., including at least one measure of CIPN symptoms, CIPN impact, and balance and gait) proposed by Park and colleagues [8], limiting our ability to compare and analyze results between studies [8]. Consistent with findings of a systematic review [35], discrepancies in the outcome measures used across the included studies hindered the ability to create a pooled effect estimate for meta-analysis. It is worth noting that 10 out of the 20 studies were published after the core outcome set was established, but may have been designed and conducted prior to publication of the outcome set paper. The heterogeneity in CIPN outcome measures underscores the need for future trials to adopt the proposed standardized core outcome set.

There are strengths and limitations to each CIPN outcome measure type, highlighting the complementary nature of using them in combination. Three studies in this review [15,22,25] relied on a single patient-reported outcome measure. Patient-reported outcome measures capture subjective symptom burden and health-related quality of life, are patient-relevant, inexpensive, and easy to implement [8]. Because patient-reported outcome measures are subjective, they may be influenced by day-to-day factors such as mood and symptom flare-ups. Furthermore, patient-reported outcome measures are susceptible to individual biases, such as social desirability bias [9]. According to one study [37], patient-reported and functional CIPN measures do not correlate well with clinical measures such as quantitative sensory testing and neurological examinations. Despite this lack of correlation, nine studies in this review did not include a clinical measure [15,16,22,23,25,29,30,33,34]. Objective clinical measures can provide important neurological and physiological input, yet are costly and require specialized equipment and training to implement [8]. Furthermore, these clinical measures do not accurately assess the functional impact of CIPN [8]. Functional outcome measures are practical and assess exercise-related movements and actions [8]. Functional measures can also capture aspects not reflected by commonly used patient-reported outcome measures, such as physical performance and sensory ataxia [8]. Thus, the combined use of patient-reported, functional, and clinical outcome measures addresses many of the limitations of each type, consistent with previous reports [8].

Our findings suggest significant variability in the effects of exercise on CIPN symptoms. When prescribing exercise to individuals with cancer, the FITT principle can be used to enhance understanding of exercise efficacy, facilitate interpretation and comparison of study results, and inform the application of research findings in practice [38,39]. The FITT principle is an acronym representing exercise Frequency, Intensity, Time, and Type, providing clarity and a standardized approach to exercise prescriptions [38]. No two studies that demonstrated significant reductions in CIPN were consistent in their FITT parameters, reflecting the current ambiguity in exercise prescription for CIPN. Although the ESMO-EONS-EANO guideline on therapy-induced neurotoxicity recommends exercise to reduce CIPN symptoms, the most recent Exercise Guidelines for Cancer Survivors consensus statement does not include recommendations for implementing exercise as a potential intervention for CIPN [40,41]. Therefore, further research is needed to establish a standardized set of FITT parameters for CIPN. Only one study in this review [19] initiated the exercise intervention before the participants began chemotherapy. While no significant differences were found between the intervention and control groups following the intervention, further research is needed to explore exercise as a potential preventive measure against CIPN [42].

A large discrepancy exists in the representation of cancer types in the studies included in this review. Breast, lung, and colorectal cancers were the most commonly reported in this review, limiting generalizability to other cancer types [43]. Although 77% of participants in the studies were female, there is little evidence to support differences in CIPN onset or maintenance by sex [44]. The chemotherapy agent administered to study participants varied across studies. CIPN presentations can differ by chemotherapy agent [45], making it challenging to conduct inter-study comparisons. Thus, future studies should consider subgroup analyses for cancer and chemotherapy types, where possible, to account for potential differing effects of exercise interventions on CIPN symptoms.

Limitations of this scoping review include the lack of studies incorporating the core outcome set and modest inter-rater agreement during screening (k = 0.55). As our primary goal in this scoping review was to gauge the landscape of outcome measures used to assess exercise effectiveness for CIPN, we considered both completed studies, secondary analyses, and protocols for ongoing studies to broaden the range of outcome measures captured, ensuring a complete representation of current and emerging practices. Due to resource constraints and a lack of end user input, the consultation stage of the Arksey and O’Malley framework [10], as refined by Levac and colleagues [11], was not conducted in this scoping review. Moreover, our review findings indicate that few studies implemented long-term follow-ups after the end of the intervention period. Thus, there is limited exploration of the long-term effects of sustained exercise on CIPN, and longitudinal studies are warranted to further investigate this area.

## 5. Conclusions

**Figure 2:**
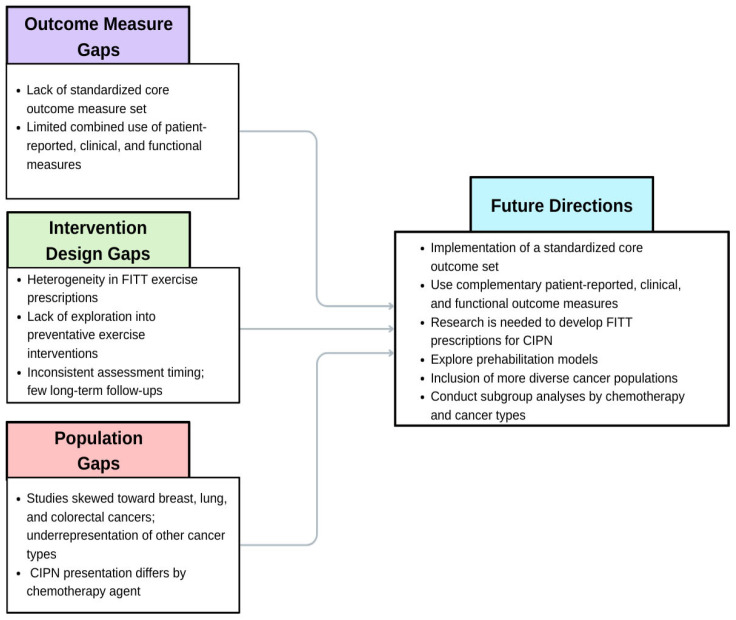
Gaps and Future Directions.

In studies that implement exercise interventions for CIPN, selecting appropriate outcome measures is crucial for tracking CIPN symptoms and their effects. We summarized gaps and future directions in Figure 2. Although a core outcome measure set for CIPN has been proposed, no studies to date have met all criteria of this recommended set, resulting in substantial heterogeneity and limiting cross-study comparability. The combined use of patient-reported, functional, and clinical outcome measures addresses the limitations inherent in any single approach. Further research is needed to clarify the effects of exercise on CIPN and to establish standardized FITT parameters for each exercise type. Furthermore, investigating exercise as a potential preventive measure against CIPN before chemotherapy initiation may be warranted. To account for variability across chemotherapy and cancer types, future studies should include subgroup analyses. As CIPN often persists in individuals following chemotherapy completion, longitudinal exercise studies with extended long-term follow-ups are warranted.

## Data Availability

No data were created or analyzed in this study. Data sharing is not applicable to this article.

## Supplementary Materials

The following supporting information can be downloaded at https://www.mdpi.com/article/10.3390/curroncol33040231/s1, Preferred Reporting Items for Systematic reviews and Meta-Analyses extension forScoping Reviews (PRISMA-ScR) Checklist.
