# Supplementary material for: Outcome Measures to Assess the Effectiveness of Exercise Interventions on Chemotherapy-Induced Peripheral Neuropathy (CIPN): A Scoping Review
Source: Curr Oncol. 2026 Apr 20;33(4):231. doi: 10.3390/curroncol33040231 (PMC13114490; doi:10.3390/curroncol33040231)
Supplement: Supplementary file 1 [file curroncol-33-00231-s001.zip › curroncol-4232633-supplementary.pdf]

Preferred Reporting Items for Systematic reviews and Meta-Analyses extension for Scoping Reviews (PRISMA-ScR) Checklist

SECTION	ITEM	PRISMA-ScR CHECKLIST ITEM	REPORTED ON PAGE #
**TITLE**			
Title	1	Identify the report as a scoping review.	1
**ABSTRACT**			
Structured summary	2	Provide a structured summary that includes (as applicable): background, objectives, eligibility criteria, sources of evidence, charting methods, results, and conclusions that relate to the review questions and objectives.	1
**INTRODUCTION**			
Rationale	3	Describe the rationale for the review in the context of what is already known. Explain why the review questions/objectives lend themselves to a scoping review approach.	2-3
Objectives	4	Provide an explicit statement of the questions and objectives being addressed with reference to their key elements (e.g., population or participants, concepts, and context) or other relevant key elements used to conceptualize the review questions and/or objectives.	3
**METHODS**			
Protocol and registration	5	Indicate whether a review protocol exists; state if and where it can be accessed (e.g., a Web address); and if available, provide registration information, including the registration number.	3
Eligibility criteria	6	Specify characteristics of the sources of evidence used as eligibility criteria (e.g., years considered, language, and publication status), and provide a rationale.	3
Information sources*	7	Describe all information sources in the search (e.g., databases with dates of coverage and contact with authors to identify additional sources), as well as the date the most recent search was executed.	3
Search	8	Present the full electronic search strategy for at least 1 database, including any limits used, such that it could be repeated.	Appendix I
Selection of sources of evidence†	9	State the process for selecting sources of evidence (i.e., screening and eligibility) included in the scoping review.	3-4
Data charting process‡	10	Describe the methods of charting data from the included sources of evidence (e.g., calibrated forms or forms that have been tested by the team before their use, and whether data charting was done independently or in duplicate) and any processes for obtaining and confirming data from investigators.	4
Data items	11	List and define all variables for which data were sought and any assumptions and simplifications made.	4
Critical appraisal of individual sources of evidence§	12	If done, provide a rationale for conducting a critical appraisal of included sources of evidence; describe the methods used and how this information was used in any data synthesis (if appropriate).	N/A

SECTION	ITEM	PRISMA-ScR CHECKLIST ITEM	REPORTED ON PAGE #
Synthesis of results	13	Describe the methods of handling and summarizing the data that were charted.	4
**RESULTS**			
Selection of sources of evidence	14	Give numbers of sources of evidence screened, assessed for eligibility, and included in the review, with reasons for exclusions at each stage, ideally using a flow diagram.	4
Characteristics of sources of evidence	15	For each source of evidence, present characteristics for which data were charted and provide the citations.	5
Critical appraisal within sources of evidence	16	If done, present data on critical appraisal of included sources of evidence (see item 12).	N/A
Results of individual sources of evidence	17	For each included source of evidence, present the relevant data that were charted that relate to the review questions and objectives.	4-13
Synthesis of results	18	Summarize and/or present the charting results as they relate to the review questions and objectives.	8-13
**DISCUSSION**			
Summary of evidence	19	Summarize the main results (including an overview of concepts, themes, and types of evidence available), link to the review questions and objectives, and consider the relevance to key groups.	14-15
Limitations	20	Discuss the limitations of the scoping review process.	15
Conclusions	21	Provide a general interpretation of the results with respect to the review questions and objectives, as well as potential implications and/or next steps.	15-16
**FUNDING**			
Funding	22	Describe sources of funding for the included sources of evidence, as well as sources of funding for the scoping review. Describe the role of the funders of the scoping review.	16

JBI = Joanna Briggs Institute; PRISMA-ScR = Preferred Reporting Items for Systematic reviews and Meta-Analyses extension for Scoping Reviews.
* Where *sources of evidence* (see second footnote) are compiled from, such as bibliographic databases, social media platforms, and Web sites.
† A more inclusive/heterogeneous term used to account for the different types of evidence or data sources (e.g., quantitative and/or qualitative research, expert opinion, and policy documents) that may be eligible in a scoping review as opposed to only studies. This is not to be confused with *information sources* (see first footnote).
‡ The frameworks by Arksey and O'Malley (6) and Levac and colleagues (7) and the JBI guidance (4, 5) refer to the process of data extraction in a scoping review as data charting.
§ The process of systematically examining research evidence to assess its validity, results, and relevance before using it to inform a decision. This term is used for items 12 and 19 instead of "risk of bias" (which is more applicable to systematic reviews of interventions) to include and acknowledge the various sources of evidence that may be used in a scoping review (e.g., quantitative and/or qualitative research, expert opinion, and policy document).

From: Tricco AC, Lillie E, Zarin W, O'Brien KK, Colquhoun H, Levac D, et al. PRISMA Extension for Scoping Reviews (PRISMAScR): Checklist and Explanation. Ann Intern Med. 2018;169:467–473. doi: 10.7326/M18-0850.

Medline, Embase, CINAHL, Scopus

Ovid MEDLINE(R) ALL <1946 to January 16, 2026>
Date searched: Jan 19, 2026
Results: 433

https://login.ezproxy.library.ualberta.ca/login?url=http://ovidsp.ovid.com/ovidweb.cgi?T=JS&NEWS=N&PAGE=main&SHAREDSEARCHID=2ee7LzvetsHkjotfx2TGfyEzXiY2KkQIFtJwGX9CZIB9I5FTEhr5Z8p5dPkCriz63

1 peripheral nerve injuries/ 8863
2 (Peripheral neuropath* or cipn or ((numbness or tingling) adj3 (hand or hands or fingers or foot or feet or toes or ankle* or extremities))).mp. 32103
3 1 or 2 40789
4 exp antineoplastic agents/ or exp angiogenesis inhibitors/ or exp antibiotics, antineoplastic/ or exp anticarcinogenic agents/ or exp antimetabolites, antineoplastic/ or exp antimitotic agents/ or exp antineoplastic agents, alkylating/ or exp antineoplastic agents, hormonal/ or exp antineoplastic agents, phytogenic/ or exp antineoplastic agents, immunological/ or exp myeloablative agonists/ or exp "poly(adp-ribose) polymerase inhibitors"/ or exp topoisomerase inhibitors/ 1367547
5 (chemotherap* or antineoplastic or taxane*).mp. 1006720
6 exp Neoplasms/ 4200045
7 (oncolog* or neoplas* or carcinom* or tumor* or tumour* or cancer* or malignan* or hemato-oncological or leukemi* or leukaemi* or AML or lymphom* or hodgkin* or T-cell or B-cell or non-hodgkin* or nephroblastom* or neuroblastom* or rhabdomyosarcom* or teratom* or hepatom* or hepatoblastom* or medulloblastom* or PNET* or meningiom* or gliom*).mp. 6079581
8 4 or 5 or 6 or 7 6872021
9 physical therapy modalities/ or exp exercise therapy/ 114110
10 (exercise* or physical activity or aerobic* or workout* or fitness program or kinesiotherap* or "core stability" or "resistance activit*" or weightlifting or (lift* adj3 weight*) or progressive resist* or "free weight*" or gravity resistive or isotonic or isometric or ((eccentric or concentric) adj2 contraction*) or (train* adj4 (balance or resist* or strength* or weight or stability or circuit* or interval* or endurance or sensorimotor or vibration)) or (musc* adj4 (strengthen* or train* or contraction*)) or (activat* adj4 muscle*) or treadmill* or ((conditioning or strengthening) adj5 (program* or protocol* or regime* or strateg* or therap* or intervention* or progressive or functional or post-operative or postoperative or home or home-based or outpatient)) or (cardio* adj3 (class* or exercis* or train* or machine*)) or ergometer* or ergometre* or walking or ((walk or walks) adj3 (fast or brisk* or quickly or regular or program*)) or swim* or running or jogging or cycling or bicycl* or bike or biking or spinning or "spin class*" or "spin bike*" or "step count*" or "count step*" or calisthenics or aquacise or aquaaerobic* or aquafit or aquasize or Fitness-tracker* or acceleromet* or pedomet* or heart-rate-monitor* or fitbit* or apple-watch* or garmin or gps or "global positioning system*").mp. 1302706

11 ("physical therap*" or physiotherap* or kinesiotherap*).mp. 102145
12 9 or 10 or 11 1375074
13 3 and 8 and 12 523
14 ((child* or pediatric* or paediatric* or adolesc* or youth or youths or teen*) not adult*).ti. 1380280
15 13 not 14 497
16 limit 15 to animals 74
17 limit 16 to humans 17
18 15 not (16 not 17) 440
19 (rat or rats or mouse or mice or murine or animal model*).ti,kf. 1691751
20 18 not 19 433

Embase <1974 to 2026 January 14> (OVID interface)
Date searched: Jan 19, 2026
Results: 755

https://login.ezproxy.library.ualberta.ca/login?url=http://ovidsp.ovid.com/ovidweb.cgi?T=JS&NEWS=N&PAGE=main&SHAREDSEARCHID=4SfwymBzOrvqjzYqXIZF0DQq9JbN9itGuAgNFOSPtr2ICrGR3UWUOz2pGfjeBs1Wq

1 (Peripheral neuropath* or cipn or ((numbness or tingling) adj3 (hand or hands or fingers or foot or feet or toes or ankle* or extremities))).tw. 51771
2 exp cancer chemotherapy/ 696906
3 exp antineoplastic agent/ 3580192
4 exp neoplasm/6670666
5 (chemotherap* or antineoplastic or taxane*).mp. 1710075
6 (oncolog* or neoplas* or carcinom* or tumor* or tumour* or cancer* or malignan* or hemato-oncological or leukemi* or leukaemi* or AML or lymphom* or hodgkin* or T-cell or B-cell or non-hodgkin* or nephroblastom* or neuroblastom* or rhabdomyosarcom* or teratom* or hepatom* or hepatoblastom* or medulloblastom* or PNET* or meningiom* or gliom*).mp. 8463827
7 2 or 3 or 4 or 5 or 6 10382755
8 physiotherapy/134766
9 exp kinesiotherapy/ 127385
10 (exercise* or physical activity or aerobic* or workout* or fitness program or kinesiotherap* or "core stability" or "resistance activit*" or weightlifting or (lift* adj3 weight*) or progressive resist* or "free weight*" or gravity resistive or isotonic or isometric or ((eccentric or concentric) adj2 contraction*) or (train* adj4 (balance or resist* or strength* or weight or stability or circuit* or interval* or endurance or sensorimotor or vibration)) or (musc* adj4 (strengthen* or train* or contraction*)) or (activat* adj4 muscle*) or treadmill* or ((conditioning or strengthening) adj5 (program* or protocol* or regime* or strateg* or therap* or intervention* or progressive or functional or post-operative or postoperative or home or home-based or outpatient)) or (cardio*

adj3 (class* or exercis* or train* or machine*)) or ergometer* or ergometre* or walking or ((walk or walks) adj3 (fast or brisk* or quickly or regular or program*)) or swim* or running or jogging or cycling or bicycl* or bike or biking or spinning or "spin class*" or "spin bike*" or "step count*" or "count step*" or calisthenics or aquacise or aquaaerobic* or aquafit or aquasize or Fitness-tracker* or acceleromet* or pedomet* or heart-rate-monitor* or fitbit* or apple-watch* or garmin or gps or "global positioning system*").mp. 1897020
11 ("physical therap*" or physiotherap* or kinesiotherap*).mp. 234899
12 8 or 9 or 10 or 11 2034193
13 1 and 7 and 12 1584
14 ((child* or pediatric* or paediatric* or adolesc* or youth or youths or teen*) not adult*).ti. 1715301
15 13 not 14 1510
16 limit 15 to animal studies 85
17 limit 16 to human 19
18 15 not (16 not 17) 1444
19 (rat or rats or mouse or mice or murine or animal model*).ti,kf. 1957732
20 18 not 19 1432
21 limit 20 to conference abstracts 519
22 20 not 21 913
23 limit 22 to "clinical trials (clinicaltrials.gov)" 158
24 22 not 23 755

CINAHL Plus with Full Text (EBSCOhost interface)
Date searched: Jan 19, 2026
Results: 245

S1 ("Peripheral neuropath*" or cipn or ((numbness or tingling) N3 (hand or hands or fingers or foot or feet or toes or ankle* or extremities)))

S2 (MH "Neoplasms+") or (MH "Antineoplastic Agents+") or (chemotherap* or antineoplastic or taxane* or oncolog* or neoplas* or carcinom* or tumor* or tumour* or cancer* or malignan* or hemato-oncological or leukemi* or leukaemi* or AML or lymphom* or hodgkin* or T-cell or B-cell or non-hodgkin* or nephroblastom* or neuroblastom* or rhabdomyosarcom* or teratom* or hepatom* or hepatoblastom* or medulloblastom* or PNET* or meningiom* or gliom*)

S3 (MH "Physical Therapy") OR (MH "Therapeutic Exercise+") or ("physical therap*" or physiotherap* or kinesiotherap* or exercise* or "physical activity" or aerobic* or workout* or "fitness program*" or kinesiotherap* or "core stability" or "resistance activit*" or weightlifting or (lift* N3 weight*) or "progressive resist*" or "free weight*" or "gravity resistive" or isotonic or isometric or ((eccentric or concentric) N2 contraction*) or (train* N4 (balance or resist* or strength* or weight or stability or circuit* or interval* or endurance or sensorimotor or vibration)) or (musc* N4 (strengthen* or train* or contraction*)) or (activat* N4 muscle*) or treadmill* or ((conditioning or strengthening) N5 (program* or protocol* or regime* or strateg* or therap* or intervention* or progressive or functional or post-operative or postoperative or home or home-based or outpatient)) or (cardio* N3 (class* or exercis* or train* or machine*)) or ergometer* or

ergometre* or walking or ((walk or walks) N3 (fast or brisk* or quickly or regular or program*)) or swim* or running or jogging or cycling or bicycl* or bike or biking or spinning or "spin class*" or "spin bike*" or "step count*" or "count step*" or calisthenics or aquacise or aquaaerobic* or aquafit or aquasize or Fitness-tracker* or acceleromet* or pedomet* or heart-rate-monitor* or fitbit* or apple-watch* or garmin or gps or "global positioning system*")

S4 TI((child* or pediatric* or paediatric* or adolesc* or youth or youths or teen*) not adult*) OR TI(rat or rats or mouse or mice or murine or "animal model*")

S5 (S1 AND S2 AND S3) NOT S4

Scopus (Advanced search)
Date searched: Jan 19, 2026
Results: 695

(TITLE-ABS("Peripheral neuropath*" or cipn or ((numbness or tingling) W/3 (hand or hands or fingers or foot or feet or toes or ankle* or extremities))) AND TITLE-ABS-KEY(chemotherap* or antineoplastic or taxane* or oncolog* or neoplas* or carcinom* or tumor* or tumour* or cancer* or malignan* or hemato-oncological or leukemi* or leukaemi* or AML or lymphom* or hodgkin* or T-cell or B-cell or non-hodgkin* or nephroblastom* or neuroblastom* or rhabdomyosarcom* or teratom* or hepatom* or hepatoblastom* or medulloblastom* or PNET* or meningiom* or gliom*) AND TITLE-ABS-KEY("physical therap*" or physiotherap* or kinesiotherap* or exercise* or "physical activity" or aerobic* or workout* or "fitness program*" or kinesiotherap* or "core stability" or "resistance activit*" or weightlifting or (lift* W/3 weight*) or "progressive resist*" or "free weight*" or "gravity resistive" or isotonic or isometric or ((eccentric or concentric) W/2 contraction*) or (train* W/4 (balance or resist* or strength* or weight or stability or circuit* or interval* or endurance or sensorimotor or vibration)) or (musc* W/4 (strengthen* or train* or contraction*)) or (activat* W/4 muscle*) or treadmill* or ((conditioning or strengthening) W/5 (program* or protocol* or regime* or strateg* or therap* or intervention* or progressive or functional or post-operative or postoperative or home or home-based or outpatient)) or (cardio* W/3 (class* or exercis* or train* or machine*)) or ergometer* or ergometre* or walking or ((walk or walks) W/3 (fast or brisk* or quickly or regular or program*)) or swim* or running or jogging or cycling or bicycl* or bike or biking or spinning or "spin class*" or "spin bike*" or "step count*" or "count step*" or calisthenics or aquacise or aquaaerobic* or aquafit or aquasize or Fitness-tracker* or acceleromet* or pedomet* or heart-rate-monitor* or fitbit* or apple-watch* or garmin or gps or "global positioning system*")) AND NOT (TITLE((child* or pediatric* or paediatric* or adolesc* or youth or youths or teen*) AND NOT adult*) OR TITLE(rat or rats or mouse or mice or murine or "animal model*"))
